# Resource Optimization Techniques and Security Levels for Wireless Sensor Networks Based on the ARSy Framework

**DOI:** 10.3390/s18051594

**Published:** 2018-05-17

**Authors:** Jumadi Mabe Parenreng, Akio Kitagawa

**Affiliations:** 1 Electrical Engineering and Computer Science, Kanazawa University, Ishikawa 920-1192, Japan; 2 Informatic Engineering and Computer Education, State University of Makassar, Makassar 90222, South of Sulawesi, Indonesia

**Keywords:** Wireless Sensor Networks, ARSy framework, resource aware, security aware, security levels, mining data, adaptable system

## Abstract

Wireless Sensor Networks (WSNs) with limited battery, central processing units (CPUs), and memory resources are a widely implemented technology for early warning detection systems. The main advantage of WSNs is their ability to be deployed in areas that are difficult to access by humans. In such areas, regular maintenance may be impossible; therefore, WSN devices must utilize their limited resources to operate for as long as possible, but longer operations require maintenance. One method of maintenance is to apply a resource adaptation policy when a system reaches a critical threshold. This study discusses the application of a security level adaptation model, such as an ARSy Framework, for using resources more efficiently. A single node comprising a Raspberry Pi 3 Model B and a DS18B20 temperature sensor were tested in a laboratory under normal and stressful conditions. The result shows that under normal conditions, the system operates approximately three times longer than under stressful conditions. Maintaining the stability of the resources also enables the security level of a network’s data output to stay at a high or medium level.

## 1. Introduction

Wireless Sensor Networks (WSNs) enable the monitoring and controlling of the physical environment from a remote location with high accuracy. Supporting technology is applied to various domains such as monitoring of environmental, agricultural, healthcare, public-safety, military, industrial, transportation systems [1], and smart homes for appliances [2]. Early detection of natural disasters, such as tsunamis, earthquakes, landslides, flash floods, fires, and hurricanes, is very important. The main supporting technology for early-warning-detection systems involves integrating sensors into a network, which can be wired or wireless.

WSNs are usually composed of components that are smaller than those in wired networks, low-cost, and applicable for a wide range of applications. The main components are sensing, processing, communication, and power units [3]. With these WSNs, it is possible to collect, process, and analyze data and send the results; therefore, their placement can be flexible [4]. These advantages make WSNs very powerful; however, they also have many limitations, such as dependence on batteries as energy sources, smaller central processing unit (CPU) and memory capacity [5], security vulnerabilities [6,7], and radio inference [8,9]. Because of these limitations, a resource-aware policy [10,11,12,13] for adaptation mechanisms and security-aware policy [14,15,16,17] for data security based on the available resources of sensor nodes are required.

The placement of a sensor node is not always in an area that is easy to reach and sometimes placed in extremely dangerous areas [18]. It is difficult to maintain a sensor node that has been deployed to monitor an area where routine maintenance, such as changing batteries, cannot be undertaken. Hence, WSNs must operate over long periods, and their resources must be used as efficiently as possible [3]. The regulation of energy consumption via adaptation is a method for increasing the lifetime of a sensor node [5,18,19].

This study is a continuation of our previous research in which we proposed an Adaptable Resource and Security (ARSy) Framework [20], which implements resource and security adaptations. Resource adaptation is used to manage battery, CPU, and memory use of a sensor node, and security adaptation is used to implement a certain security level to compensate for the excessive use of resources. Security adaptation works on the basis of resource availability. If the average use of a resource is above a critical threshold, the security of the generated data is at a high or medium level; however, if resource availability decreases beyond the threshold, the security of such data is at a low level or even very low level.

The remainder of this paper is as follows. In Section 2, we discuss related studies. In Section 3, we discuss the requirements and architecture of a security system and its adaptation mechanisms and processes. In Section 4, we discuss the laboratory testing we conducted and the results. In Section 5, we provide discussion and present the conclusion and direction for future study in Section 6.

## 2. Related Studies

There were challenges in previous studies on data processing in a sensor node because data mining was executed on data streams [10,11]. Online and real-time data storage and processing are executed with limited computing capabilities [12,21,22]. To overcome energy inefficiency in WSNs, the first solution is to reduce the amount of data communication by moving the data-processing algorithm to the sensor network, and the second solution is to combine data-processing and communication units [13].

Data mining is required to reduce the amount of data that must be transmitted through data communications [22,23]. There are many algorithms for mining data such as clustering [24], classification [25], and frequent items [26]. To avoid massive data transfers, a sensor node is implemented for on-board analysis, with which data are processed at the source location. Previous projects, such as VEDAS [27], EVE [28], and Diamond Eye [29], have used this method.

Security systems offer as much protection as possible; thus, power consumption will increase and the lifetime of the system will decrease. System services are reduced to decrease power consumption, which decreases the lifetime of the system [30]. In fact, security is almost always higher than potential threats. When security is very strong, it affects the overall performance of the system, excessive protection will reduce reliability and availability and affect security globally. An appropriate level of security can be estimated in terms of providing different security-quality protection models for each type of data [31].

The concept of green security [32] or re-engineering of security [30] can be an alternative solution, but it requires time and money to implement. Secure smart home concept using Home Area Network (HAN) [2], and Home Automation System (HAS) using Virtual Machines [8] make it easier to manage the security system. Previous studies focused on a security policy to model the security level of data that may have different outputs generated over time because determining the security level of data is based on the availability of resources. With a better availability of resources, the security level of data becomes higher [14,20].

The absolute requirement of a security system is the guarantee of high data security; however, in cases in which WSNs are used, high data security affects the performance and lifetime of the system because a higher data-security level means greater energy consumption for cryptographic data functions [3,33,34]. The solution is to balance the use of resources through the security level of data [14,15], which is basically used to offset the use of resources when their availability has entered a critical phase. The policy of applying a high level of security to each output affects the lifetime of a WSN because higher security levels of data put greater demands on the CPU and increase battery consumption [33].

## 3. System Design

Initially, hardware systems were designed with embedded sensor devices to guarantee the compatibility of the sensor and system. The largest obstacle is finding a device that can integrate the battery, memory, CPU, and communication units. Large-capacity CPU and memory are required because data processing is done on-board. After reviewing several types of components, a Raspberry Pi 3 Model B and DS18B20 temperature sensor were chosen for our laboratory testing.

The Raspberry Pi 3 Model B was chosen because it is a single-board model, simple, and lightweight. The model has built-in Wi-Fi, eliminating the need for extra USB Wi-Fi adapters [35]. Another advantage is its compatibility with several operating systems and its plug-and-play compatibility with a variety of equipment. The specifications for this model are listed in Table 1.

Devices with the Raspberry Pi 3 Model B pose challenges. One challenge involves memory sharing between a CPU and graphic processing unit (GPU) [37]. Some programs are not as demanding on the CPU, and some also run on the GPU such as Blu-ray video playback. A GPU is powerful enough to handle applications. The second challenge is with the power-supply-management system.

The DS18B20 temperature sensor is a single-wire digital sensor [36] that uses only one cable for communication with the CPU and for grounding. The sensor can derive power directly from the data line. The specifications are also listed in Table 1.

### 3.1. Architecture System

**Figure 1:**
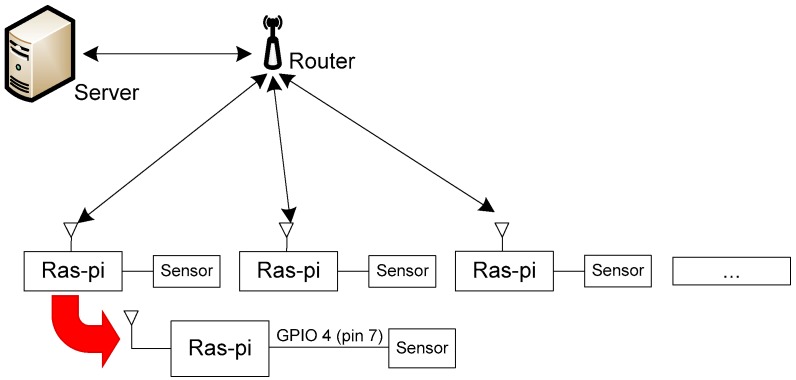
Architecture system.

We conducted our laboratory testing on a single node; however, future work will involve integrating the node with a wider network system, such as the architecture system shown in Figure 1. The data collected by each node are processed with local node resources in accordance with the conditions of the resource node. The output data are sent at certain times to the server, which is the final destination of the data.

### 3.2. ARSy Framework

**Figure 2:**
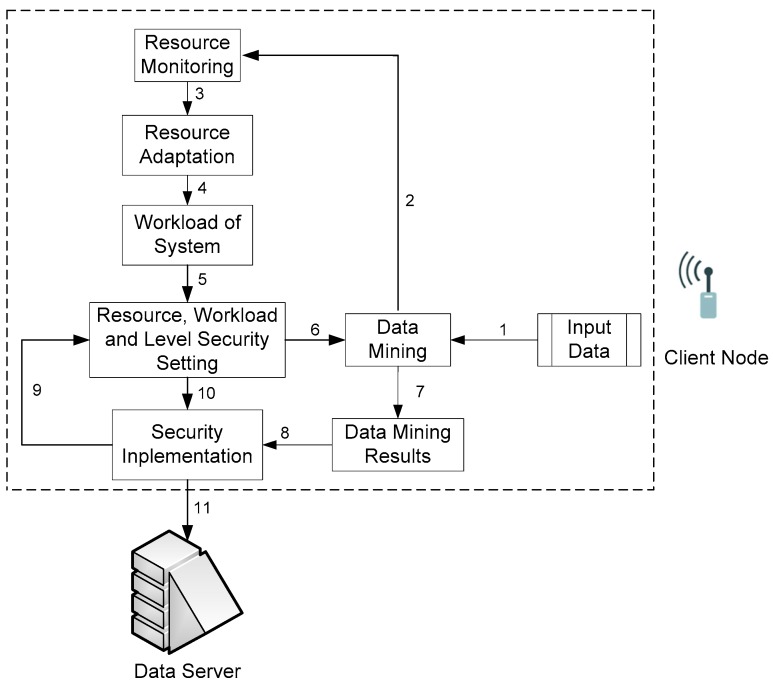
ARSy Framework.

Generally, our ARSy framework [20] consists of two parts, i.e., a client node, which applies the method of collecting data on-board [28], and a data server, which stores sensor-node results, as shown in Figure 2.

The client node is a worker node. The process that occurs on the client node is divided into the following process blocks. Details of the relationship between the resources and security level of this process are listed in Section 3.2.2.

- *Data-input block*: This block collects data. The collection time was every second in our study. Before the data are processed by the *data-mining block*, the system checks the conditions of the battery, CPU and memory resources in the *resource-monitoring block*.
- *Resource-monitoring block*: This block reports the latest update of the average amount of the sensor node’s resources. This information is then input for the *resource-adaptation block*.
- *Resource-adaptation block*: This block updates the resource condition with two modes, the first is the status of the resource under adaptation conditions and the second is the status of the resource not under such conditions.
- *Workload-system block*: This block provides the workload status of the sensor node if the resources system has a heavy or light workload; heavy workload status if information resources received from resource adaptation blocks under adaptation conditions and light workload status if resource information from the *resource-adaptation block* is not under adaptation conditions.
- *Resource-, workload-, and security-level-setting block*: This block contains the summary of the all the system’s resource conditions, such as amount of resources that can be used to execute data mining, security-level status that will be given at data output, and overall system workload.
- *Data-mining block*: This block involves mining data based on light-weight frequent algorithm [22,38]. The data-mining process is carried out by creating counter data. All the same data are placed on the same counter data and new counter data are created for new data types.
- *Data-mining-result block*: This block temporarily stores the results of data mining whose time-limited, before the data-mining-result sent to the *Security-implementation block*.
- *Security-implementation block*: This block implements the appropriate security level based on the resource-sensor-node condition, then the results are sent to the data server, i.e., the final destination of all data.

#### 3.2.1. Resource Adaptation

The battery, CPU, and memory are the main resources; therefore, resource availability must be maintained. This is achieved through adaptation [11,12], whereas the parameters data and process are maintained based on our ARSy framework. Each resource in a security system is limited by the critical threshold. If the threshold is exceeded, adaptation will be triggered to reduce excessive resource usage.

We divided resource adaptation of the sensor node into three mechanisms, i.e., *input*, *process*, and *output*. Input adaptation is triggered using the battery resource, process adaptation is triggered using the CPU resource, and output adaptation is triggered using the memory resource. The resource-adaptation formulas are listed in Table 2.

When an adaptation occurs in one of the sensor-node resources by applying the specific adaptation policy to that resource (see Table 2). These mechanisms are described in more detail as follows.

- *Input*: Battery adaptation is triggered on the input side and is based on battery resource availability via the sampling interval (SI) [10,12]. If the battery usage exceeded the threshold, adaptation will be triggered. Before battery-resource adaptation occurs, the input-data-collection time is normal (does not exceed the threshold), but adaptation is triggered when the input-data-collection time changes based on the available resources.
- *Process*: CPU adaptation is triggered on the process-data side and is based the availability of the processor resource (CPU) via the random factor (RF) [10,12]. If the CPU usage does not exceed the threshold, all the collected data will be maintained on the counter data; otherwise, the system only stores some counter data with priority based on dominant and non-dominant counter data. Some non-dominant counter data will be eliminated to relieve the CPU; its value will be based on the RF value.
- *Output*: Memory adaptation is triggered on the output-data side and is based on the availability of the memory resource via the radius threshold (RT) [10,12]. When the resource memory is normal (not exceeding the threshold), the final result is sent to the data server; as much as 50% of all counter data is saved. If the memory usage exceeds the threshold, the system will reduce memory utilization by limiting the amount of counter data created, and the counter data are stored as output data based on the RT presentation value.

#### 3.2.2. Security Adaptation

The adaptation of resources affects the security level of data. The security-adaptation model we applied is for estimating the security level of output data based on the resource condition. When the resource condition does not exceed the threshold, the output data have the maximum security level, but when resource availability falls below the threshold, the security level changes [20], as shown in Table 3.

## 4. Results

Our testing was limited to single node, and the goal was to observe the differences in resource behavior and time efficiency when a security system operates under normal and stressful conditions and implementing an ARSy framework and a non-ARSy framework.

**Figure 3:**
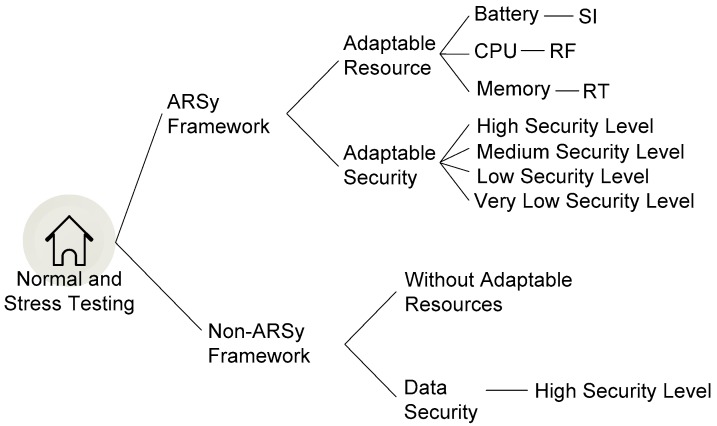
Testing design.

The testing design is illustrated in Figure 3. Testing with the ARSy framework involved combining resource adaptation and security adaptation in the security system, while testing with the non-ARSy framework involved resources that do not adapt under critical conditions and all security output data are generated with a high security level.

### 4.1. Testing Scenarios

We tested under two scenarios: normal and stress. Normal testing was conducted by allowing the system to run normally without any intervention or special treatment that would cause the CPU to become busier than usual. Stress testing was conducted by making the CPU busier, such as by playing games, browsing websites, and streaming videos. The sensor was touched so that variants data could be collected; otherwise, there would be too few data variants. Stress testing continued until the resources were completely exhausted. The uniform testing parameters are listed in Table 4.

Critical threshold of battery, CPU, and memory: The resource used exceeded the threshold and triggered resource adaptation. Data capture: the time of collecting data was every second. Release data: The data results to be sent to the data server, the time of which was set according to the requirement testing. Battery capacity: The battery capacity differed depending on the test, as shown in Table 5. Testing scenarios: Normal and stress. Sensor treatment: because we used a temperature sensor, then to see the variation of data capture, treatment with the sensor is touched and untouched to show the data-mining functions for variants data captured, as described in Section 4.3.

### 4.2. Resources

The resource activities of a WSN are interrelated. In general, battery consumption is strongly affected by the activity of the CPU, i.e., busy, normal, or idle. Increased CPU activity increases battery consumption [33].

#### 4.2.1. Battery

**Figure 4:**
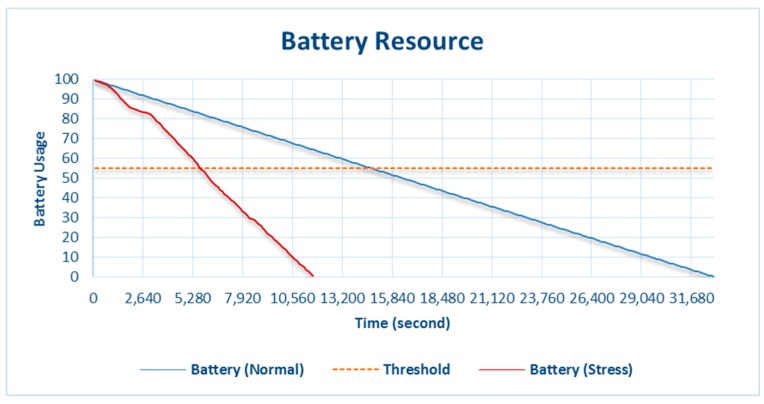
Battery consumption during normal and stress testing.

**Figure 5:**
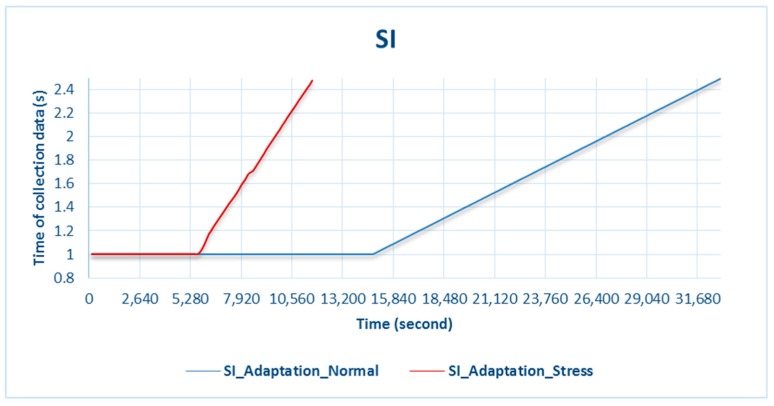
Battery adaptation during normal and stress testing.

Figure 4 shows the battery slowly entering the critical phase and exceeding the threshold until the battery is completely discharged. The system’s policy before the battery exceeded the threshold significantly affected the sensor’s data-input process. Data were collected every second when the battery was not in the adaptation phase, as shown in Figure 5, and gradually changed as the battery entered the critical phase. Adaptation of the battery through the input data affected the data-collection time. Under normal battery-resource availability, the data-collection time was 1 s per datum and gradually changed when the availability of the battery resource was running low.

As shown in Figure 5, when the adaptation began to trigger battery consumption, data collection gradually decreased because data input did not occur every second, but was based on the SI adaptation value. The time between one instance of data collection and the next was up to 2.5 s. When the data-collection time changed, CPU performance became more lightweight.

#### 4.2.2. CPU

**Figure 6:**
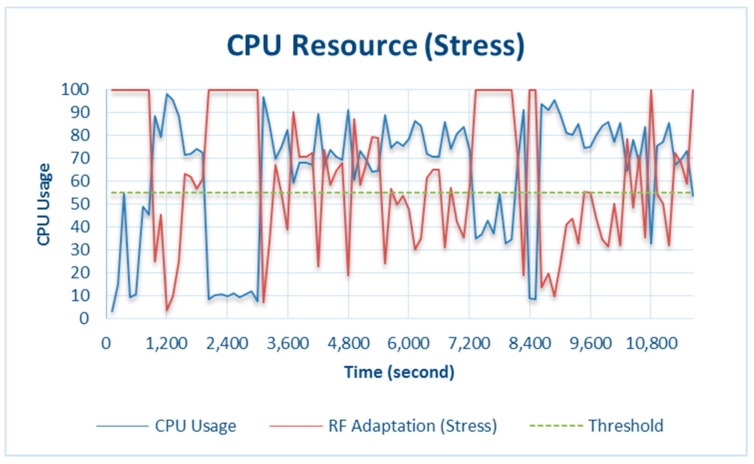
CPU resource and adaptation during stress testing.

**Figure 7:**
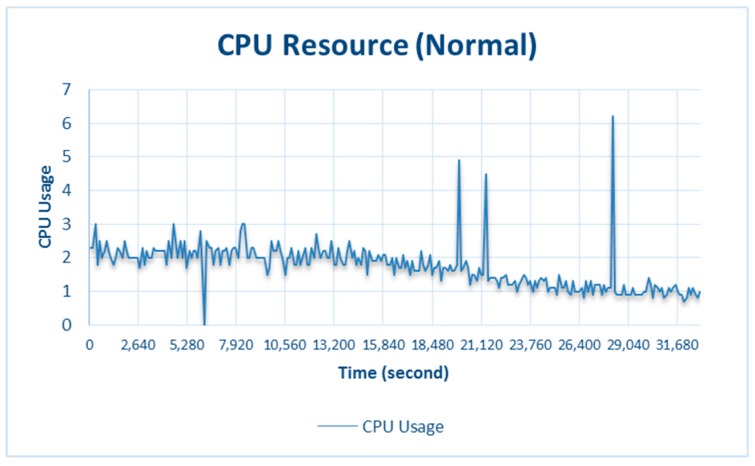
CPU resource during normal testing.

The results from testing under CPU stress conditions are shown in Figure 6. Testing was conducted by making the CPU busier than usual by playing games, browsing websites, and streaming videos. This was done because Raspberry Pi 3 Model B has a large CPU capacity (see Table 1). Testing was conducted by allowing the CPU to run normally, and the results indicate that the CPU activity never exceeded the threshold, as shown in Figure 7.

When the CPU did not exceed the threshold, the RF did not initiate adaptation; therefore, all the collected data were processed. However, when resource availability decreased below the threshold under critical conditions, CPU adaptation through the RF was triggered on the basis of the amount of CPU used, as shown in Figure 6. In 7200 s, the *CPU_used* capacity was 73.6% and CPU adaptation was 58.6%. This means that the CPU only processed 58.6% of the total data on the counter. Another condition at 7320 s was when *CPU_used* was 35.2% and CPU adaptation (RF) was 100%, i.e., the CPU processed all the data. In general, when system is in the adaptation phase, all the data collected through data mining will not be stored, but only some of the data that have more dominant data variants than others. For more details, see Section 3.2.1. Resource Adaptation.

The results shown in Figure 7 are for normal testing, where CPU_used was greatest at 6.2% at 28,200 s. This did not exceed the 55% critical threshold, so the system was not in the resource-adaptation phase.

#### 4.2.3. Memory

**Figure 8:**
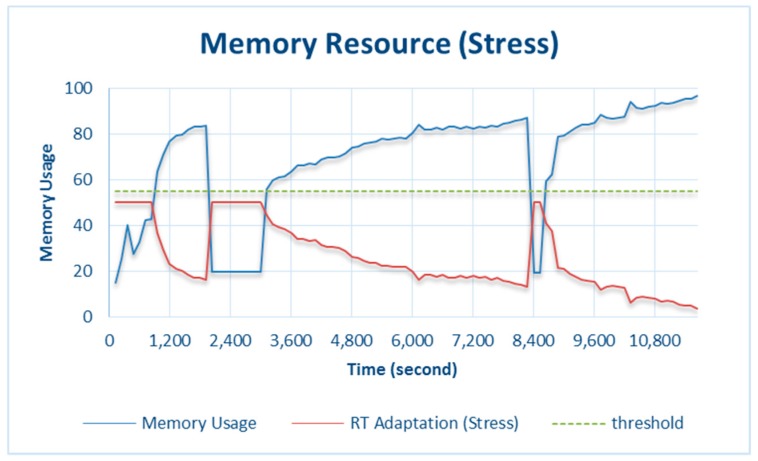
Memory resource and adaptation during stress testing.

**Figure 9:**
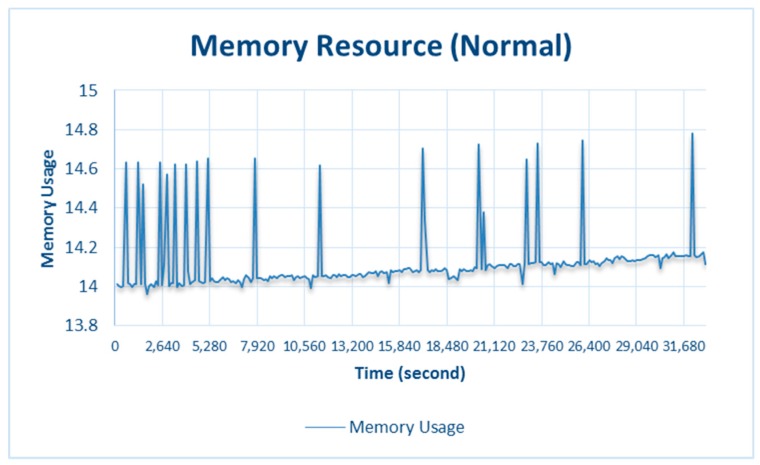
Memory resource during normal testing.

As mentioned above, the memory of Raspberry Pi 3 Model B is shared by the CPU and GPU. It is very difficult to monitor the memory exceeding the threshold value, so the procedure for testing the CPU was also conducted for the memory. Figure 8 and Figure 9 show the results of the stress and normal testing of the memory, respectively.

As shown in Figure 8, the memory initially ran normally without adaptation because usage did not yet exceed the threshold. Under this condition, the data were processed by the memory and stored. The applied system policy adaptations will save 50% of the most dominant counter data, which are the final data sent to the server. However, when the memory usage exceeded the threshold, the amount of final data sent to the data server was calculated on the basis of the value of memory adaptation through the RT.

For example, at 2400 s, *memory_usage* was 19.5%, which means no adaptation was triggered and the default *RT adaptation* value was 50%, so half the total amount of counter data was saved and sent to the data server. Furthermore, at 6000 s, *memory_usage* was 80.4%, which exceeded the threshold, so adaptation was triggered. The data to be stored constituted 19.6%.

Figure 9 shows the results of memory usage during normal testing. Memory usage at 32,160 s, resulted in memory_usage being 14.78%, which did not exceed the memory threshold, so memory adaptation was not triggered.

### 4.3. Collecting and Mining Data

Data collected by the sensors are processed by an on-board mechanism [27,28,29], i.e., by directly applying a data-mining algorithm known as the Lightweight Frequent Item Algorithm [20,22,38]. The data are placed on the counter data based on the similarity of the data and new counter data are created if the collected data differ from those previously formed on the counter data. This data-mining process continues until the time limit is reached.

**Figure 10:**
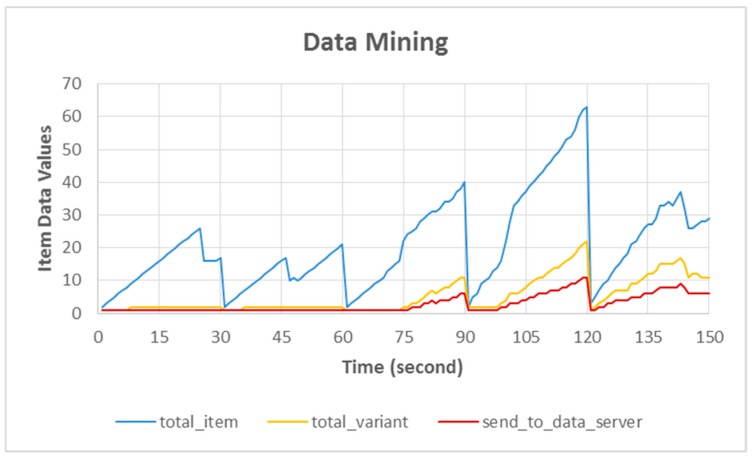
Collecting and mining data.

Figure 10 compares the three types of data collected after the data-mining process, the release period of the data-mining result was every 30 s: (1) *total_item*, all data collected by sensors that were limited by the timer; (2) *total_variant,* the number of data variants obtained during the data-mining period; and (3) *send_to_server,* the final data to be sent to the data server.

For example, the data in this graph are at 120 s, *total_item* 63 data, *total_variant* 22 data, and *send_to_server* 11 variant data. This graph is based on our ARSy framework and shows the difference between *total_item, total_variant,* and *send_to_server*. During data mining, a large amount of data were captured by the sensor (*total_item 63*), but the data-mining mechanism filters by grouping similar collected data (*varian_data 22*). For the final result after processing (*send_to_server 11*), only some of the data with the most variants were sent to the data server as the final result due to the adaptation policy of the system.

### 4.4. Security Level

Determining the security level [15] is the last stage before data are sent to the data server. There are four security levels based on the availability of resources (see Table 3): level 3 is high security level, which is the most ideal because the output data sent to the data server have the maximum level of security, with the average resource availability of the network being 75–100%; level 2 is medium security level with the average resource availability being 50–75%; level 1 is low security level with average resource availability being 20–50%; and level 0 is very-low security level with average resource availability being 0–20%.

**Figure 11:**
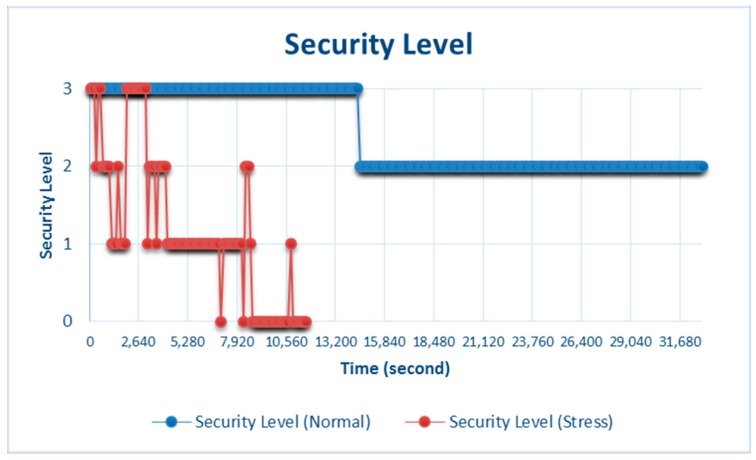
Security level of data output during stress and normal testing.

Figure 11 shows the security levels during stress and normal testing. During stress testing, the security level of the data output fluctuated and reflected the current conditions. Normal testing showed more stable results at the high and medium security levels. Hence, maintaining resource stability can also stabilize the security level at the maximum average condition.

### 4.5. Operating Time

Time operation is the duration or the length of operation time that measured in second units. Battery consumption [39] was divided into several modes, such as boot, idle, video playback, normal operation, and stress. The testing was conducted on an ARSy framework and a non-ARSy framework under normal and stress operating conditions. The results are listed in Table 5. The battery capacities were 30, 100, and 1000-mAh and release times were 30, 60, and 120 s. Details of this testing scenario are summarized in Table 4. With the ARSy framework for 1000-mAh battery capacity during normal testing, the operating duration reached 9.19 h, whereas during stress testing, the duration reached only 2.76 h. With the non-ARSy framework for 1000-mAh battery capacity during normal testing, the duration reached 3.44 h, and during stress testing, the duration reached 2.74 h.

## 5. Discussion

The availability of resources and data security on WSN devices is an absolute requirement, but resource capacity is very limited. The limited battery, CPU, and memory resources of WSN devices force the devices to use the resources as efficiently as possible. Security is the largest resource used when the highest security is required for the output security data. Resource-adaptation and security-adaptation solutions on sensors nodes are extremely important, particularly if they are used to monitor extreme areas where maintenance, such as replacing batteries or just checking the proper position of the device, is very difficult.

We implemented the ARSy Framework investigated in our previous study using Raspberry Pi 3 Model B and DS18B20 temperature sensor. The advantage of Raspberry Pi 3 Model B is that it has large CPU and memory capacities. With these advantages, are highly manageable of resources and allow integration of several types of sensors in one Raspberry Pi unit. The weakness of Raspberry Pi 3 Model B is sharing memory between a CPU and GPU; therefore, it was difficult for us to analyze in more detail the cost of memory during our testing, as shown in Table 6, where memory consume during operation in normal and stress. It was difficult to distinguish the causes of memory fluctuations due to the CPU or GPU processes. Another weakness Raspberry Pi 3 Model B is it consumes a large amount of energy. In this case, using a battery as an energy source becomes an option because other alternatives use energy harvesting or the main power source based on the design requirements.

Sending all collected data to the server is not a solution. This will consume the limited resources. Selecting data collected by a sensor is done through data mining. The data-mining is done on-board, each datum is collected by the sensor directly, and selection is done with the data-mining algorithm at the board node. The final result is then sent to the data server.

For security, data in this research is collected by estimating security-level based on average resources availability in sensor node. The more average resource availability, the higher the security level that will be implemented in the output data. The discussion in this research has not yet implemented cryptography.

Radio communication is also one of the main resources of a sensor node and consumes the largest amount of resources. However, in this study implement the ARSy framework, we limited resource adaptation to the battery, CPU, and memory because the implementation and testing were on a single node.

## 6. Conclusions and Future Study

The limited battery, CPU, and memory resources of WSN devices force such devices to use resources as efficiently as possible. We evaluated a security adaptation for limited resources in a WSN through the ARSy framework. By mining data on-board and applying resource and security adaptation, the operation duration can be tripled. Normal testing showed that the result is more stable at the high and medium security levels. Therefore, maintaining resource stability can also stabilize the security level under the maximum average condition. The comparison of the ARSy framework and a non-ARSy framework showed significant results during operation time.

To conserve the battery of the sensor node, harvesting energy can be the best solution, depending on the area where the system is deployed. Because our testing was conducted on a single node, for the future work testing should be conducted on several nodes integrated with a network system involving energy harvesting as the power source and implement security level based on cryptography.

## Figures and Tables

**Table 1:** Hardware system specifications.

Components	Specifications
Raspberry Pi 3 Model B [35]	Single-board, 1.2 GHz, 64-bit quad core, 1-GB RAM
	Wi-Fi, micro SD, HDMI, USB, GPIO
	Power usage 5.19 V, 2.5 A maximum
DS18B20 [36]	Single-wire digital temperature sensor
	Minimum −55 °C
	Maximum 125 °C
	Power consumption DC 3.0–5.5 V

**Table 2:** Resource-adaptation formulas.

Resource	Definition	Parameters
Battery [11,12,20]	$SI=ub-(bat\_available)*\left(\frac{ub-lb}{batt\_crit\_threshold}\right)$	*SI*: Sampling Interval, *bat_available*: free battery, *ub*: upper bound, *lb*: lower bound. *RF*: Random Factor, *cpu_crit_threshold*: critical threshold cpu, *RT*: Radius Threshold, *mem_crit_thres*: critical threshold memory.
CPU	$RF=(100-CPU\_used)*\left(\frac{ub-lb}{100-cpu\_crit\_threshold}\right)$	
Memory	$RT=(100-mem\_used)*\left(\frac{ub-lb}{100-mem\_crit\_threshold}\right)$	

**Table 3:** Relationships between resources and security level.

Resources	Workload	Security Level	Average Amount of Resources (%)
Maximum	Light	High	75–100
Maximum	Light	Medium	50–75
Minimum	Heavy	Low	20–50
Minimum	Heavy	Very Low	0–20

**Table 4:** Testing parameters.

Parameter	Value
Critical threshold of Battery, CPU, and Memory	55% capacity in use
Time for data collection	1 s
Time for data release	60 s
	120 s
Battery capacity	100 mAh
	1000 mAh
Tests	Normal
	Stress
Sensor treatment	Touched
	Untouched

**Table 5:** Operation duration.

Battery Capacity [mAh]	Release Time [s]	ARSy		Non-ARSy	
		Normal [s]	Stress [s]	Normal [s]	Stress [s]
30	30	948	249	355	223
100	60	3265	1468	1222	900
1000	120	33,085	9971	12,397	9877

**Table 6:** Memory consumption during normal and stress testing.

Time	Consumption under Normal (Bytes)		Consumption under Stress (Bytes)	
	Memory Free	Memory Consume	Memory Free	Memory Consume
2	810,692,608	(53,248)	807,817,216	1,134,592
3	810,627,072	65,536	805,052,416	2,764,800
4	810,639,360	(12,288)	799,571,968	5,480,448
5	810,614,784	24,576	797,908,992	1,662,976
6	810,532,864	81,920	793,935,872	3,973,120
…	…	…	…	…
125	809,201,664	49,152	377,528,320	1,806,336
126	809,259,008	(57,344)	380,477,440	(2,949,120)
127	809,103,360	155,648	380,395,520	81,920
128	809,250,816	(147,456)	376,307,712	4,087,808
129	809,201,664	49,152	376,213,504	94,208
…	…	…	…	…
241	808,214,528	(204,800)	221,683,712	2,981,888
242	808,194,048	20,480	219,455,488	2,228,224
243	808,202,240	(8192)	216,629,248	2,826,240
244	808,214,528	(12,288)	217,247,744	(618,496)
245	808,218,624	(4096)	213,950,464	3,297,280
